# Early Synaptic Alterations and Selective Adhesion Signaling in Hippocampal Dendritic Zones Following Organophosphate Exposure

**DOI:** 10.1038/s41598-019-42934-z

**Published:** 2019-04-25

**Authors:** Karen L. G. Farizatto, Michael F. Almeida, Ronald T. Long, Ben A. Bahr

**Affiliations:** 1 Biotechnology Research and Training Center, University of North Carolina-Pembroke, Pembroke, North Carolina USA; 2 Department of Biology, University of North Carolina-Pembroke, Pembroke, North Carolina USA; 3 Department of Chemistry and Physics, University of North Carolina-Pembroke, Pembroke, North Carolina USA

**Keywords:** Cellular neuroscience, Extracellular signalling molecules

## Abstract

Organophosphates account for many of the world’s deadliest poisons. They inhibit acetylcholinesterase causing cholinergic crises that lead to seizures and death, while survivors commonly experience long-term neurological problems. Here, we treated brain explants with the organophosphate compound paraoxon and uncovered a unique mechanism of neurotoxicity. Paraoxon-exposed hippocampal slice cultures exhibited progressive declines in synaptophysin, synapsin II, and PSD-95, whereas reduction in GluR1 was slower and NeuN and Nissl staining showed no indications of neuronal damage. The distinctive synaptotoxicity was observed in dendritic zones of CA1 and dentate gyrus. Interestingly, declines in synapsin II dendritic labeling correlated with increased staining for β1 integrin, a component of adhesion receptors that regulate synapse maintenance and plasticity. The paraoxon-induced β1 integrin response was targeted to synapses, and the two-fold increase in β1 integrin was selective as other synaptic adhesion molecules were unchanged. Additionally, β1 integrin–cofilin signaling was triggered by the exposure and correlations were found between the extent of synaptic decline and the level of β1 integrin responses. These findings identified organophosphate-mediated early and lasting synaptotoxicity which can explain delayed neurological dysfunction later in life. They also suggest that the interplay between synaptotoxic events and compensatory adhesion responses influences neuronal fate in exposed individuals.

## Introduction

Organophosphate and phosphoramidate compounds are deadly poisons that inhibit acetylcholinesterase activity, causing severe symptoms and death in exposed individuals, occasionally including first responders. These toxic nerve agents have been used in chemical warfare for many decades, eliciting a potent action on the central nervous system and resulting in a cholinergic crisis in exposed individuals^1–3^. Besides being used in warfare and acts of terrorism, organophosphate compounds have been and still are widely used as pesticides and herbicides, thus causing heightened public health concerns.

Survivors of organophosphate exposure exhibit delayed neuronal injury, chronic behavioral morbidities, and cognitive dysfunction correlating with reduced hippocampal volume^4–6^. A number of reports suggest that organophosphate exposure is associated with an increased risk for several chronic neurological illnesses^7–12^. In fact, asymptomatic low-level exposure to the nerve agent soman leaves brain tissue vulnerable to subsequent insults related to stroke and seizures^13^. Early signs for neurological disorders include pathogenic changes in synaptic components which can lead to neuronal dysfunction, as occurs in models of nerve agent exposure^13–15^. Thus, in addition to respiratory and cardiac distress that can be lethal after coming in contact with a toxic organophosphate, it is important to understand the neuronal consequences of low-level contact that underlie the neurological and behavioral abnormalities expressed by exposure survivors.

The organophosphate toxin paraoxon (Pxn) is the main active metabolite of the pesticide parathion used in the United States and other countries, in which parathion is converted to Pxn by oxidation in the soil and the liver^16,17^. Like nerve agents, Pxn causes excitotoxic brain damage through the irreversible inhibition of acetylcholinesterase^18^, affecting behavior, cognition, and may trigger seizures and elongate epileptiform activity^19–22^. The Pxn toxin and related organophosphates cause similar neurological problems, often long after an exposure^9,21–23^. Pxn also disrupts developmental processes underlying synaptic connectivity and cognitive ability in children, who appear to be particularly vulnerable to anticholinesterase effects^24,25^.

Here, Pxn was studied in hippocampal slice cultures to understand organophosphate-mediated effects that may underlie the long-term symptoms in survivors of nerve agent exposure. Cultured hippocampal slices have been widely used to study the actions of neurotoxins because they provide a stable tissue model that maintains the native organization and circuitry of the adult hippocampus and exhibits the pathogenic responsiveness found *in vivo*^13,26–32^. Sensitive indications of neuronal compromise have been found in the explant model to involve modifications to important synaptic markers including GluR1, synapsins, PSD-95, and synaptophysin^13,28,33,34^. In the present report, Pxn was infused into mature slice cultures in order to examine the pathogenic cascade associated with anticholinesterase toxicity and its effect on the profile of synaptic constituents.

## Material and Methods

### Preparation of Hippocampal Slice Cultures

This study was carried out in strict accordance with Animal Welfare Act and other Federal statutes and regulations related to animals and their use. The procedures adhered to recommendations from the Guide for the Care and Use of Laboratory Animals of the National Institutes of Health. Animal use and analyses were conducted in accordance with approved protocols of the Animal Care and Use Committees of the University of North Carolina at Pembroke (IACUC protocol number: 2015-04). Litters of Sprague-Dawley rats (Charles River Laboratories, Wilmington, MA) were housed in accordance with guidelines from the National Institutes of Health. Brain tissue from postnatal 12-day-old rats was rapidly removed to prepare hippocampal slices as described previously^26,32^. Transverse slices (400 μm thickness) were prepared and kept in cooled-buffer solution until 8–9 slices were placed on the Biopore PTFE membrane of each Millicell-CM culture insert (Millipore, Billerica, MA). Media was changed every 2–3 days, consisting of 50% basal medium Eagle (Sigma-Aldrich, St. Louis, MO), 25% Earle’s balanced salts (Sigma-Aldrich), 25% horse serum (Gemini Bio-products, Sacramento, CA), and defined supplements, as described previously^35–37^. The hippocampal slices were maintained in culture at 37 °C in 5% CO_2_-enriched atmosphere for a maturation period of 18–20 days before experiments were conducted.

### Treatment of Hippocampal Slice Cultures

Pxn (Tocris, Ellisville, MO) was routinely prepared as a 5 mM stock solution in ice-cold serum-free media, followed by immediate dilution for infusion into cultures. Groups of cultured hippocampal slices were treated with vehicle or freshly prepared 200 μM Pxn. Note that the Pxn concentration and conditions used were previously established^37^. Slice groups with Pxn exposure times of 3–48 h were staggered in order to ensure same-day harvesting, and vehicle control groups were incubated for 24–48 h. A subset of Pxn-treated hippocampal tissue was co-treated with the fatty acid amide hydrolase inhibitor AM5206, previously shown to have neuroprotectant activity against neurotoxin exposure^37^. The tissues were pre-treated for 1 h with 20 μM AM5206, and subsequently treated with Pxn with continued presence of AM5206. In another set of slice cultures, after the 24-h Pxn treatment the cultures were subjected to a procedure to washout the anticholinesterase, followed by incubating the cultures in control media for an additional 24 h. The treatment groups were offset by one day to ensure same-day harvesting. Finally, a small group of slice cultures were treated with 300 μM NMDA for 24 h to provide a positive control group that exhibits the obvious neurodegeneration induced by NMDA-mediated excitotoxicity. The cultured slices were then fixed in 4% paraformaldehyde for 24 h, or they were gently removed from the inserts with a soft brush into groups of 7–9 slices each using ice-cold isosmotic buffer containing 0.32 M sucrose, 5 mM HEPES (pH 7.4), 1 mM EDTA and 1 mM EGTA.

### Immunohistochemistry

Fixed hippocampal slices were rinsed in PBS and blocked with 5% BSA in PBS containing 0.1% TX100 for 1.5 h at room temperature. Next, the tissue was incubated overnight at 4 °C with primary antibodies against NeuN (1:200, Abcam, Cambridge, MA), synaptophysin (1:200, Abcam), βIII tubulin (1:500, Abcam), GFAP (1:1000, Abcam), synapsin II (1:200, Abcam), GluR1 (1:200, Millipore), β1 integrin (1:200, Millipore), PSD-95 (1:200, Santa Cruz Biotechnology, CA) and the HUTS-4 monoclonal antibody specific for the active conformation of β1 integrin (1:200, Millipore). The tissue slices were then rinsed three times for 10 min each and incubated with appropriate Alexa Fluor secondary antibodies (1:750, Molecular Probe, Thermo Scientific, Rockford, IL) and Neurotrace 640/660 deep-red fluorescent Nissl staining (1:100, Thermo-Scientific). After incubation and three rinses, the slices were mounted on slides and coverslips applied with mounting medium containing 4′,6-diamidino-2-phenylindole (DAPI; Vector Laboratories, Burlingame, CA) to visualize cell nuclei by confocal microscopy. The specificities of the primary antibodies were confirmed by performing immunoblots with rat and mouse brain samples. Specificity of secondary antibodies was tested in the absence of primary antibody.

Immunofluorescence analyses were performed with a Nikon C2 point-scanning confocal microscope with NIS-Elements AR software (Nikon Instruments Inc., Melville, NY). For defined neuronal and dendritic zones assessed for immunoreactivity levels, 30–40 images per channel (405, 488, 568, and 647 nm) were acquired to assemble individual data sets using a z-distance of 0.3–1 μm. Five z-stack data sets for each antigen were combined for mean area fraction profiles (binary area/measured area) of each region of interest and representative images were processed for compressed maximal intensity projection. Acquisition parameters were identical for immunostaining measures between vehicle-treated and Pxn-treated slice cultures.

To test for damaged and dead neurons, a subset of control, Pxn-treated, and NMDA-treated slice cultures were incubated with 10 µg/ml propidium iodide (PI) at 37 °C for 1 h in order to label damaged or dead neurons that have become permeant to the dye. PI fluorescence was elicited at 546 nm and recorded at 610 nm.

### Immunoblot analysis

For preparing hippocampal tissue samples for immunoblot analyses, the different experimental treatments were harvested as groups of 7–9 slice cultures each in order to provide sufficient protein levels for the immunoreactivity measures of multiple markers and also to reduce the variance that can occur in explants prepared from tissue spanning the septal to temporal poles of isolated hippocampi. The grouped slice samples were assessed by immunoblot after being stored at −80 °C. The samples were thawed and sonicated in cold lysis buffer consisting of 15 mM HEPES (pH 7.4), 0.5 mM EDTA, 0.5 mM EGTA, and a protease inhibitor cocktail (Sigma-Aldrich), followed by protein concentration determined using the Pierce BCA Protein Assay (Thermo-Scientific). Equal amounts of the protein samples were denatured for 5 min at 100 °C, separated by SDS-PAGE, and transferred to nitrocellulose membranes (Bio-Rad, Hercules, CA). Next, membranes were blocked with 5% non-fat dry milk for 1 h at room temperature, followed by incubation with primary antibodies against GluR1 (1:1000) from Millipore, actin (1:500) from Sigma-Aldrich, synapsin II (1:1000), βIII tubulin (1:1000), phospho S3-cofilin (1:300), total-cofilin (1:150), synaptophysin (1:1000), NCAM-180 (1:300), and GABA_A_ receptor β1 (1:300) from Abcam, and β1 integrin (1:1000), PSD-95 (1:300) and neurexin (1:200) from Santa Cruz. For secondary antibody steps, horseradish peroxidase-conjugated goat anti-rabbit IgG (1:10,000) and goat anti-mouse IgG (1:6,000) were from Amersham GE Healthcare (Pittsburgh, PA); these steps were performed in 2% milk and incubated for 1 h at room temperature. Development of antigens used enhanced chemiluminescent reagent, followed by antigen detection with the Amersham Imager 600 (GE Healthcare). Stained antigens were scanned at high resolution to determine integrated optical density measures using the imager software.

### Statistical analysis

The results were evaluated by linear regression analysis, unpaired *t* tests, and analyses of variance (ANOVA) using Prism software (GraphPad).

## Results

**Figure 1:**
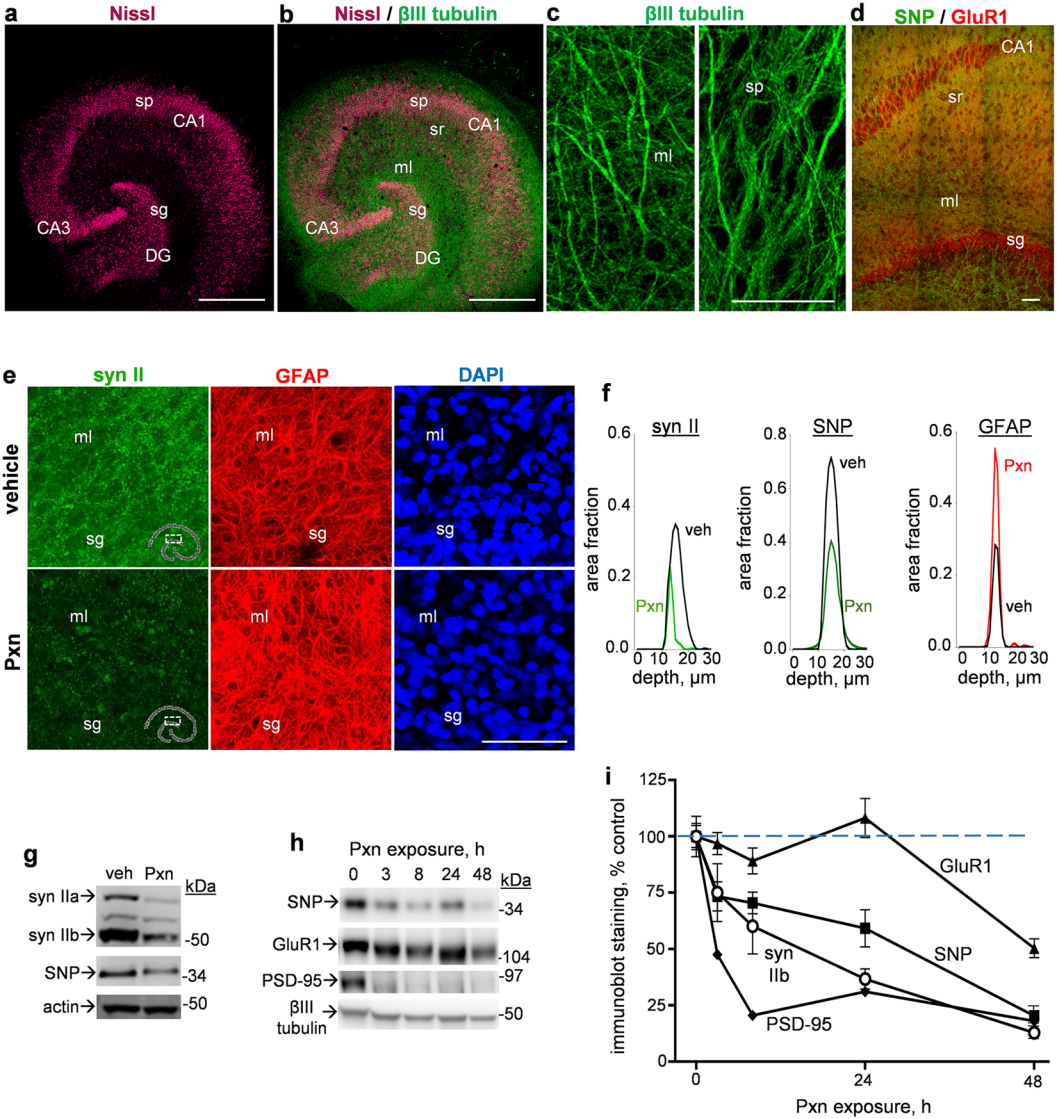
Synaptotoxicity mediated by the organophosphate Pxn was assessed in hippocampal slice cultures. Transverse slices of rat hippocampi were maintained on Biopore inserts for 18–20 days, exhibiting Nissl-stained neuronal subfields and βIII tubulin-labeled neuropil (**a**,**b**, respectively; size bar: 375 µm), as well as tubulin-positive dendritic arborization and dense co-localization of synaptic markers (**c**,**d**, respectively; size bar: 50 µm). DG, dentate gyrus; ml, molecular layer; sg, stratum granulosum; SNP, synaptophysin; sp, stratum pyramidale; sr, stratum radiatum. Slice cultures exposed to vehicle or 200 µM Pxn for 24 h were stained for synapsin II (syn II) and GFAP and images from the same view-field were processed for compressed maximal intensity projection, shown alongside DAPI staining (**e**; size bar: 50 µm). Area fraction profiles for synapsin II, synaptophysin, and GFAP from the molecular layer are shown to illustrate total immunostaining through the acquired z-stacks (**f**). Immunoblots analyzed Pxn exposure times of 0 vs. 24 h (**g**) and 0–48 h (**h**) and treatments were staggered for same-day harvesting of slice samples stained for synaptic markers, actin, and βIII tubulin (positions of molecular weight standards are shown). Full-length immunoblots are presented in Supplementary Figs S1 and S2. Immunoreactivity levels from immunoblots (percent of control; means ± SEM) were normalized to their respective 0 h control data and plotted across exposure time (**i**).

Using hippocampal tissue cultures with native cellular and neuropil features (Fig. 1a–d), we studied the toxic actions of the organophosphate Pxn. The hippocampal slice cultures indeed provide a sensitive model to study pathogenic responses^29,30,38–40^. The stratum pyramidale and stratum granulosum were assessed, as were the corresponding dendritic zones of the distinct laminar subfields. Anticholinesterase toxicity elicited by a single application of Pxn consisted of distinct reductions in synaptic proteins. As shown in Fig. 1e, the punctate staining of synapsin II was greatly reduced in the molecular layer after 24 h, and this decline was associated with increased staining for the astrocyte marker GFAP, but no change in DAPI-labeled nuclei was observed. The decline in synapsin II labeling in Pxn-treated slices paralleled that of synaptophysin across z-sections from confocal imaging (Fig. 1f), as well as in immunoblot samples (Fig. 1g, Supplementary Fig. S1) which were harvested as groups of 7–9 slice cultures each to provide sufficient material for the analysis of multiple markers. The reduced area fraction for the two presynaptic markers occurred in correspondence with a nearly two-fold increase in GFAP area staining (third graph of Fig. 1f).

Pxn-induced changes in the immunoblot measures of synaptic markers exhibited early onset, with synaptophysin and other synaptic components being reduced by 25% or more within 3 h as shown in Fig. 1h (also see Supplementary Fig. S2). The decline in synaptophysin progressed to near complete loss across the Pxn exposure profile of 3–48 h (Fig. 1i; ANOVA: p < 0.001, n = 15) as compared to no-treatment control samples (n = 12). Synapsin IIb had a comparable synaptotoxic profile with early an reduction and progressive deterioration (ANOVA: p < 0.0001, n = 16). In contrast, the Pxn-mediated reduction in the postsynaptic receptor subunit GluR1 was slow although significant (Fig. 1h,i; ANOVA: p = 0.022, n = 20). As clearly evident in Fig. 1i, the rate of the GluR1 decline was slower than the toxicity profile exhibited by synaptophysin (*F* = 10.91, p = 0.002, two-way ANOVA) and synapsin IIb (*F* = 28.18, p < 0.0001). Note that while GluR1 immunoblot levels did not correlate with those of synapsin IIb across samples of slices treated with or without Pxn for 24 h, a significant correlation was found when assessing samples treated for longer than 24 h (R = 0.969, p = 0.0001).

**Figure 2:**
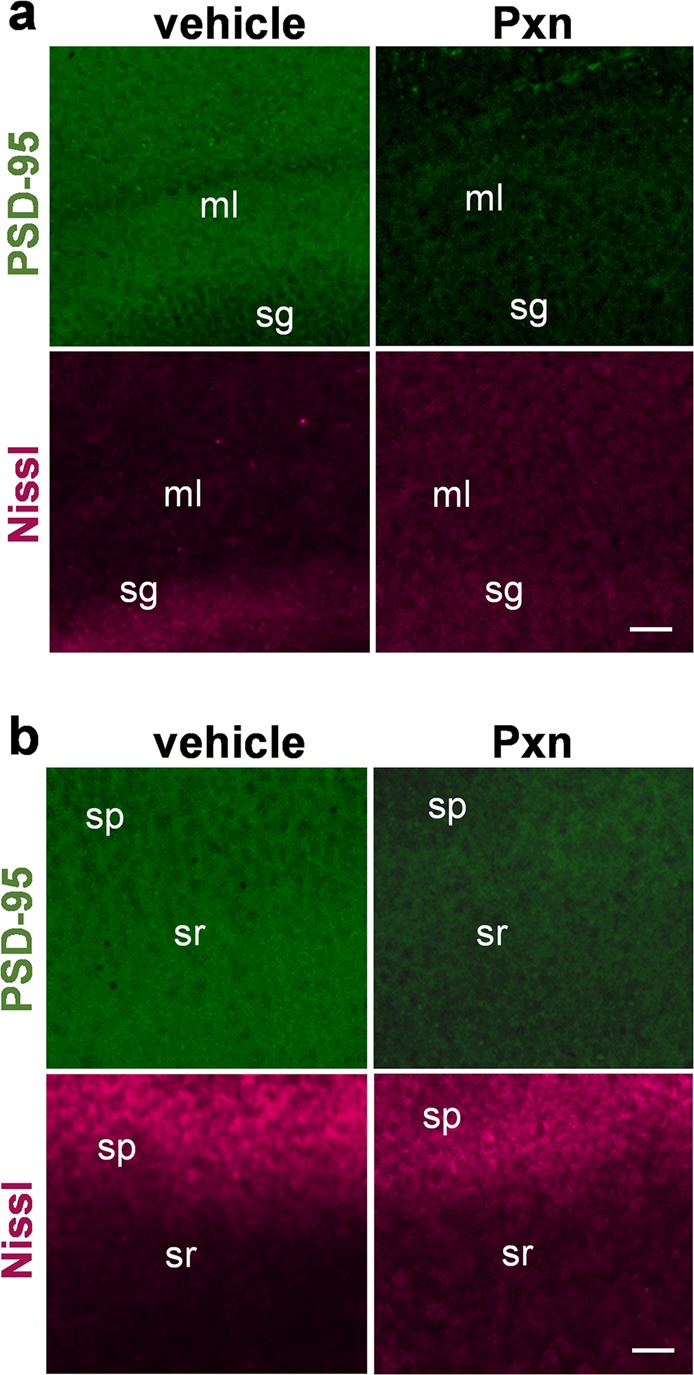
The anticholinesterase treatment results in decreased PSD-95 staining in dendritic fields of the hippocampal molecular layer (ml) and stratum radiatum (sr). Hippocampal slice cultures treated with vehicle or 200 µM Pxn for 24 h were fixed and stained for PSD-95, and images are shown with compressed maximal intensity projection alongside Nissl staining from the same view-field in the molecular layer (**a**) and the stratum radiatum (**b**). Size bar: 50 µm. sg, stratum granulosum; sp, stratum pyramidale.

Other synaptic constituents were also found reduced by Pxn, including the β1 subunit of the inhibitory GABA_A_ receptor (data not shown) and PSD-95. Similar to synaptophysin, the postsynaptic PSD-95 protein was found reduced within 3 h of Pxn exposure and exhibited a dramatic decay profile (Fig. 1h,i). Synaptophysin, synapsin IIb, and PSD-95 exhibited distinct synaptotoxicity being reduced by 70–80% after 48 h of exposure, whereas the postsynaptic GluR1 subunit was less affected by the toxin (Fig. 1h, Supplementary Fig. S2). After 72 h the exposed slice cultures exhibited 82–89% reductions in synaptophysin and synapsin IIb immunoblot measures (n = 4), a 92% reduction in PSD-95, but only a 62.5% reduction in GluR1 which is significantly smaller than the other markers (p < 0.01). In Fig. 2a images, PSD-95 tissue staining revealed a comparable level of Pxn-mediated decline in the molecular layer as described above for synapsin II. The punctate PSD-95 immunolabeling was also reduced in the CA1 stratum radiatum (Fig. 2b).

**Figure 3:**
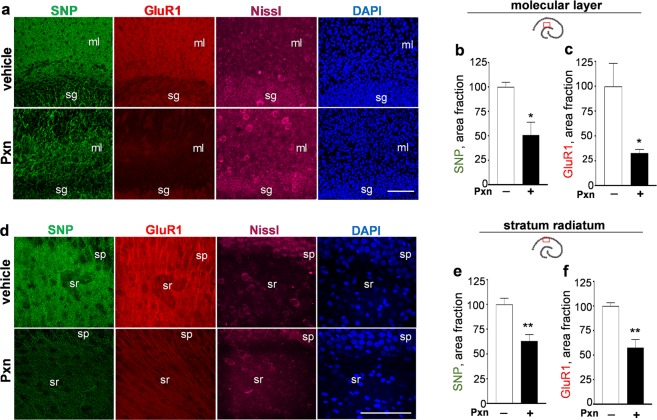
Pxn exposure reduces pre- and postsynaptic markers in hippocampal dendritic fields. Hippocampal slice cultures treated with vehicle or 200 µM Pxn for 24 h were immunostained for synaptophysin (SNP) and GluR1 and images from the same dentate view-field are shown with compressed maximal intensity projection alongside image channels for Nissl and DAPI staining (**a**). Molecular layer data from area fraction analyses of acquired z-stacks (percent of control; mean ± SEM) show Pxn-induced reductions in synaptophysin (**b**) and GluR1 (**c**). Images from the CA1 subfield are shown (**d**) and area fraction analyses of stratum radiatum data sets also found reductions in the two synaptic markers (**e,f**). Unpaired t-tests: *p < 0.05, **p < 0.01. Size bar: 100 µm. ml, molecular layer; sg, stratum granulosum; sp, stratum pyramidale; sr, stratum radiatum.

To further study the synaptotoxic profile induced by Pxn, detailed imaging by confocal microscopy was used to examine the major hippocampal subfields in control and Pxn-treated slices. Synaptic marker declines were observed in the dendritic zones of the dentate gyrus (Fig. 3a–c) and CA1 (Fig. 3d–f) that normally exhibit dense synaptic staining. The 24-h Pxn exposure caused a loss in the punctate labeling of synaptophysin as compared to vehicle-treated control slices, with area fraction analyses displaying a 50% reduction in the molecular layer (Fig. 3b) and a 37% reduction in the stratum radiatum (Fig. 3e; n = 5 for the vehicle group of slices, n = 5 for the Pxn group). In contrast to its immunoblot results, GluR1 exhibited reduced area fraction dendritic staining in the molecular layer (reduced by 67%, Fig. 3a,c) and in the stratum radiatum (reduced by 42%, Fig. 3d,f), in correspondence with the counterstained synaptophysin levels.

**Figure 4:**
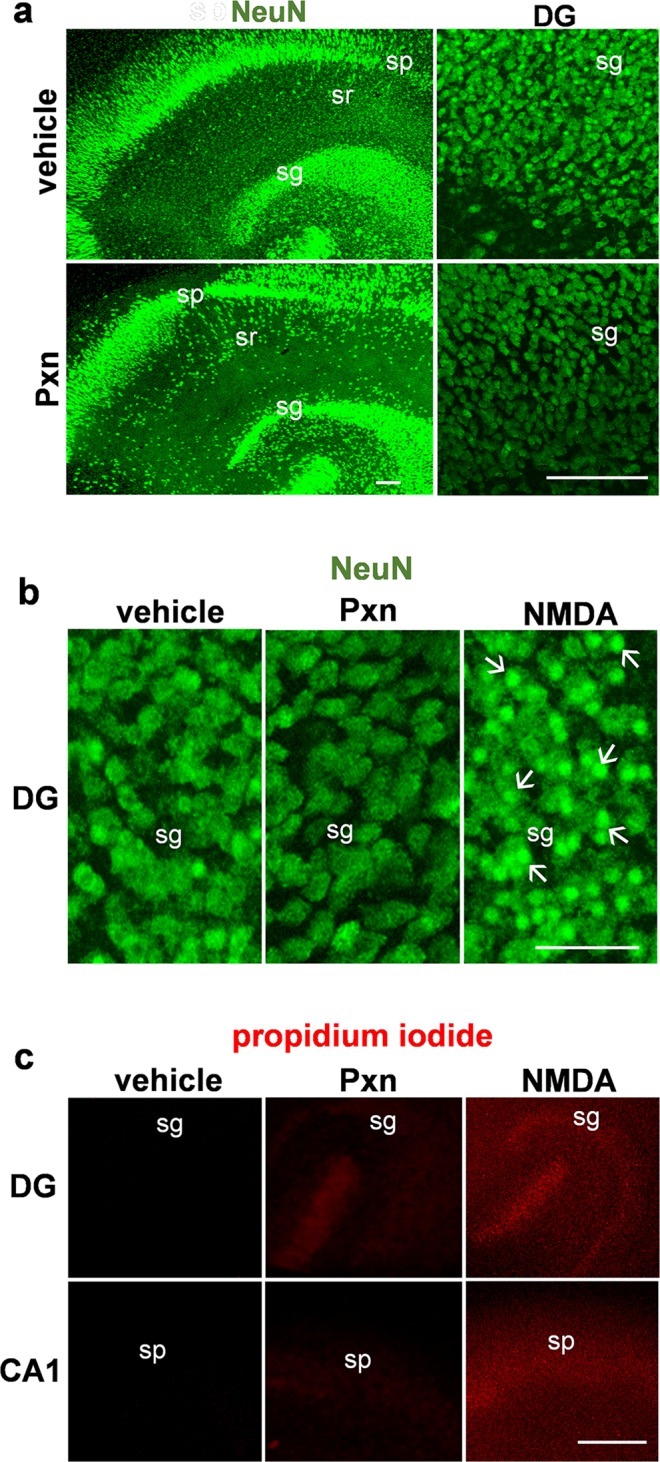
NeuN and propidium iodide staining found no indications of Pxn-induced neuronal damage. Hippocampal slice cultures were treated with vehicle, 200 µM Pxn, or 300 µM NMDA for 24 h. Fixed tissue was immunostained for NeuN and images are shown with compressed maximal intensity projection (**a**, size bar: 100 µm) as well as with high magnification views (**b**, size bar: 50 µm) to assess for neurons with pyknotic changes (see arrows). A subset of the explants were incubated with propidium iodide for 1 h and images show that only the NMDA-treated slices exhibit extensive staining of granule and pyramidal neurons, indicative of cellular damage (**c**; size bar: 250 µm). DG, dentate gyrus; sg, stratum granulosum; sp, stratum pyramidale; sr, stratum radiatum.

Note that the loss of synaptic marker immunostaining occurred in the absence of any overt changes in neuronal density or morphology in Nissl-stained tissue (see Figs 2a,b and 3a,d). As also shown in Fig. 4a for vehicle- and Pxn-treated slice cultures, NeuN immunohistochemistry revealed comparable levels of densely populated neurons in the stratum pyramidale and granule cells in the dentate gyrus. Close examination found no indications of Pxn-induced morphological alterations among NeuN-positive neurons (Fig. 4a,b). Slice samples were also assessed for NeuN by immunoblot (not shown) and the immunoreactivity measures indicated no change after 3–24 h of Pxn exposure (93.0 ± 3.0% of control, mean ± SEM) nor after 48–72 h of exposure (100.3 ± 1.4% of control). Tissue staining with anti-NeuN and propidium iodide (PI) compared the Pxn-treated slices with those subjected to NMDA-mediated excitotoxicity. In contrast to the normal granule neurons observed in Pxn slices, the NMDA-treated slices exhibited extensive pyknotic changes seen in the NeuN immunostaining (Fig. 4b). PI also extensively stained granule as well as pyramidal neurons after the NMDA insult, indicating the compromised plasma membrane permeability associated with cellular degeneration (Fig. 4c). Pxn-treated tissue, on the other hand, was absent of PI staining in the pyramidal layer and only trace amounts of labeling in the dentate gyrus.

**Figure 5:**
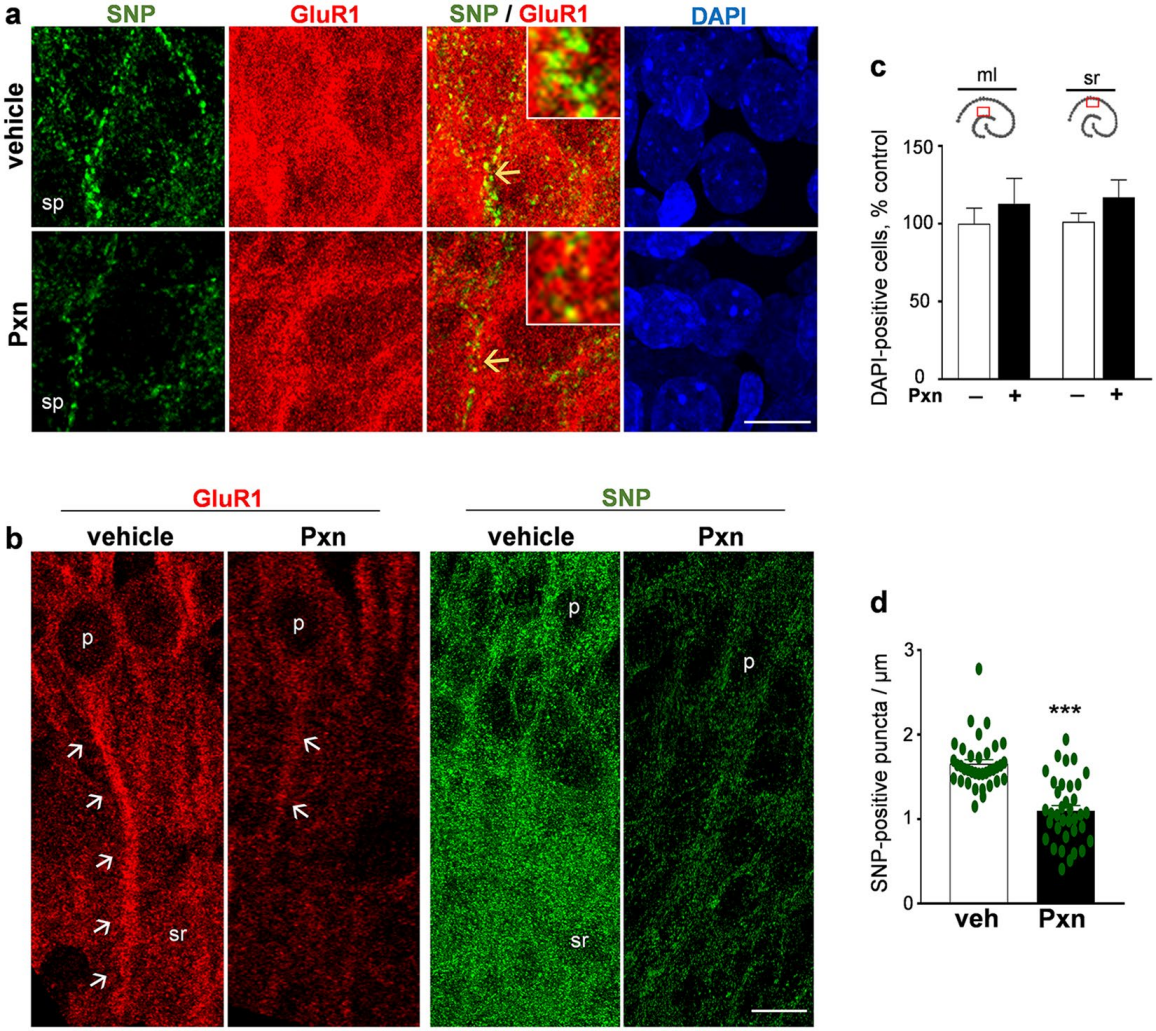
Pxn treatment causes synaptic declines in the pyramidal zone and stratum radiatum. Hippocampal slices were treated with vehicle (veh) or 200 µM Pxn for 24 h and compressed maximal intensity images were acquired from the stratum pyramidale (sp), showing reduced levels of synaptophysin (SNP) but little change in GluR1 and DAPI staining (**a**). Changes along proximal dendrites are evident in the merged images and innervated zones noted by yellow arrows are magnified in the inserts (view-width: 2.6 µm). Images extending further into the stratum radiatum (sr) were acquired to assess synaptic marker declines (**b**), notably for the arrowed GluR1 staining along apical dendrites extending from the soma of pyramidal neurons (p). Pxn treatment did not alter the number of DAPI-positive cells in either the molecular layer (ml) or stratum radiatum (**c**; percent of control, mean ± SEM). The number of synaptophysin-positive puncta was determined along innervated zones proximal to individual pyramidal neurons (**d**; mean numbers of labeled puncta per μm of dendritic zone assessed). Unpaired t-test: ***p < 0.0001. Size bar: 10 µm for (**a**,**b**).

At high magnification, the Pxn-induced loss of punctate synaptophysin labeling around and proximal to CA1 pyramidal cell bodies was more obvious than changes in the GluR1 staining (Fig. 5a). On the other hand, both synaptic markers displayed striking declines in their immunostaining in the neighboring stratum radiatum (Fig. 5b). The dense array of anti-synaptophysin punctate labeling was uniformly reduced in intensity, and the anti-GluR1 reactivity along apical dendrites was particularly decreased in the Pxn slices. To determine if the Pxn effect identified in dendritic fields is synapse-specific, DAPI-positive cells were counted in both stratum radiatum and molecular layer images (Fig. 5c) and no changes were found in either area. Using methods to detect toxicity at the synaptic level^34,41^, the number of synaptophysin-positive puncta were counted along lengths of innervated zones proximal to individual pyramidal neurons (Fig. 5d; n = 36 per group), resulting in a 34% reduction produced by the Pxn treatment (p < 0.0001). Interestingly, no reduction was found when the number of clearly defined GluR1-positive puncta were assessed for each pyramidal neuron (73 ± 20 per control neuron, mean ± s.d., n = 36 vs. 86 ± 24 per Pxn neuron, n = 35).

**Figure 6:**
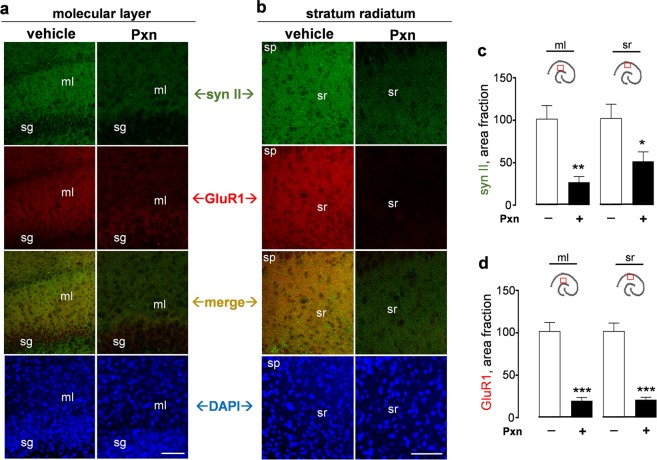
Treatment with the anticholinesterase causes corresponding declines in GluR1 and synapsin II in dendritic fields of the hippocampus. Slice cultures were exposed to vehicle or 200 µM Pxn for 24 h. Fixed tissue was assessed for synapsin II (syn II), GluR1, and DAPI and the immunostained images were processed for compressed maximal intensity projections from view-fields selected in the molecular layer (**a**) and the stratum radiatum (**b**). Size bars: 50 µm. ml, molecular layer; sg, stratum granulosum; sp, stratum pyramidale; sr, stratum radiatum. The acquired images of individual synapsin II (**c**) and GluR1 (**d**) data sets were subjected to area fraction analyses and results are shown (percent of control; mean ± SEM). Unpaired t-tests: *p < 0.05, **p < 0.01, ***p < 0.001.

With respect to the reduced GluR1 staining in the molecular layer (Fig. 6a) and stratum radiatum (Fig. 6b) after anticholinesterase exposure, further assessment found a correspondence with reduced levels of synapsin II, a synaptic vesicle-associated protein involved in the regulation of the active vesicle pool for neurotransmitter release^42^. The immunolabeling measures of synapsin II (Fig. 6c) and GluR1 (Fig. 6d) were reduced by 73–80% in the molecular layer. In the dendritic zone of the stratum radiatum, synapsin II staining decreased by 51% (Fig. 6c) and GluR1 by nearly 80% in the same confocal view-fields (Fig. 6d). The two synaptic markers exhibited correlative declines across the dendritic areas assessed (R = 0.600, p < 0.01). As with previous morphological assessments by Nissl and NeuN staining, the density and morphology of DAPI-labeled nuclei were unchanged in the dentate gyrus (Fig. 6a) and CA1 areas (Fig. 6b), further indicating the Pxn-mediated loss in synaptic markers occurs prior to cellular deterioration.

**Figure 7:**
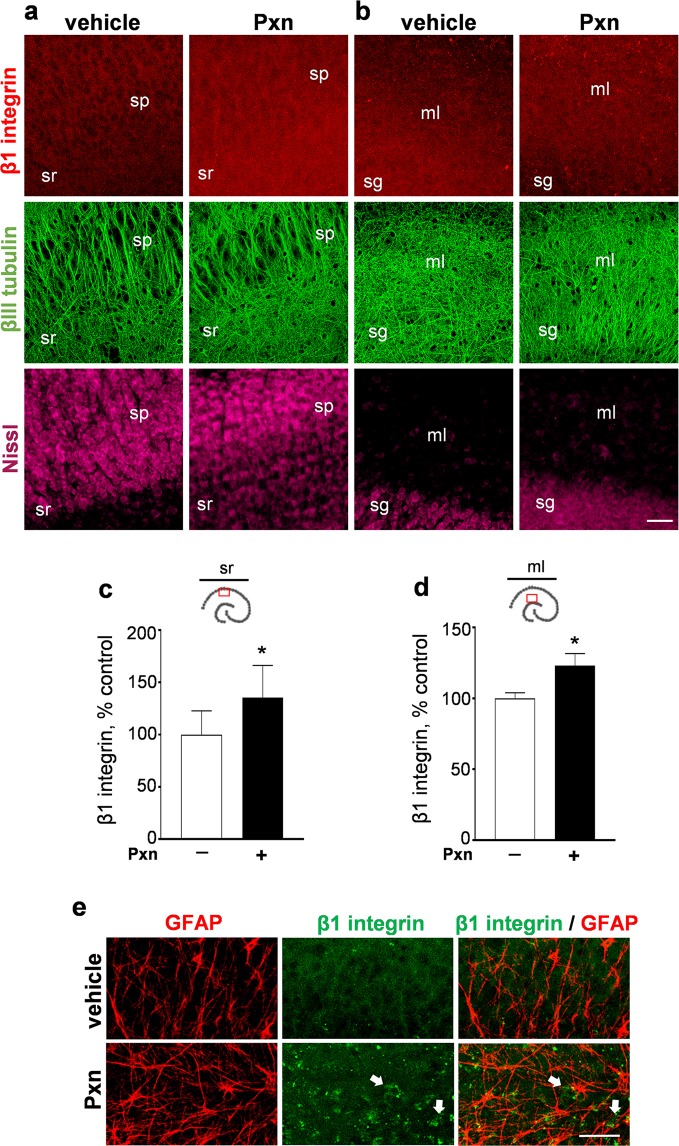
Pxn exposure increases β1 integrin labeling in hippocampal dendritic fields. Slice cultures were treated with vehicle or 200 µM Pxn for 24 h and subsequently processed for immunocytochemistry. The tissue was assessed for β1 integrin, βIII tubulin, and Nissl staining, with images processed for compressed maximal intensity projections shown for view-fields in the CA1 subfield (**a**) and the dentate gyrus (**b**). Size bar: 50 µm. ml, molecular layer; sg, stratum granulosum; sp, stratum pyramidale; sr, stratum radiatum. The acquired images of individual β1 integrin data sets from the stratum radiatum (**c**) and dentate gyrus molecular layer (**d**) were subjected to area fraction analyses and results are shown (percent of control; mean ± SEM). Unpaired t-tests: *p < 0.05. The treated slices were also double-stained for GFAP and β1 integrin (**e**, size bar: 25 µm), with images from the molecular layer indicating that the Pxn-induced β1 integrin labeling (arrows) does not coincide with GFAP-positive astrocytes.

The striking loss of synaptic markers in the dendritic zones was also found not to be due to dendritic pathology since the density of dendrites, assessed by immunohistochemical staining for the neuron-specific βIII tubulin, was unchanged (Fig. 7a,b). Assessing the immunoblot samples for Pxn exposures of 24–72 h, βIII tubulin levels were found to be 89.2 ± 6.0% of control (n = 7; N.S.). In the view-fields shown in Fig. 7, there were no indications of i) altered dendrites, ii) change in density of Nissl-stained neurons, or iii) altered neuronal morphology. However, an increase in staining was evident for the β1 integrin subunit. The β1 integrin is a matrix receptor component whose transduction of signals between the extracellular matrix and the actin cytoskeleton has been implicated in neuronal plasticity^43–46^ and the stabilization of synaptic signaling^47–50^. Opposite to the synaptic decline that Pxn produces in the stratum radiatum, β1 integrin immunolabeling was augmented (Fig. 7a), with a significant increase found by area fraction analysis (Fig. 7c). Similarly, the β1 integrin response was also evident in the molecular layer of the dentate gyrus (Fig. 7b) and the significant integrin modulation was confirmed by the analysis of area fractions (Fig. 7d). While β1 integrin responses to pathogenic insults have been shown to occur in both neurons and astrocytes^51,52^, the response in Pxn-treated slices does not show co-labeling with GFAP-positive astrocytes (Fig. 7e). Also, the distinct increase in astrocytic staining was nearly four times the modest increase in β1 integrin area fraction measures in the neuropil. The evident integrin dynamics in Pxn slices entails β1 integrin labeled neurons and punctate staining.

**Figure 8:**
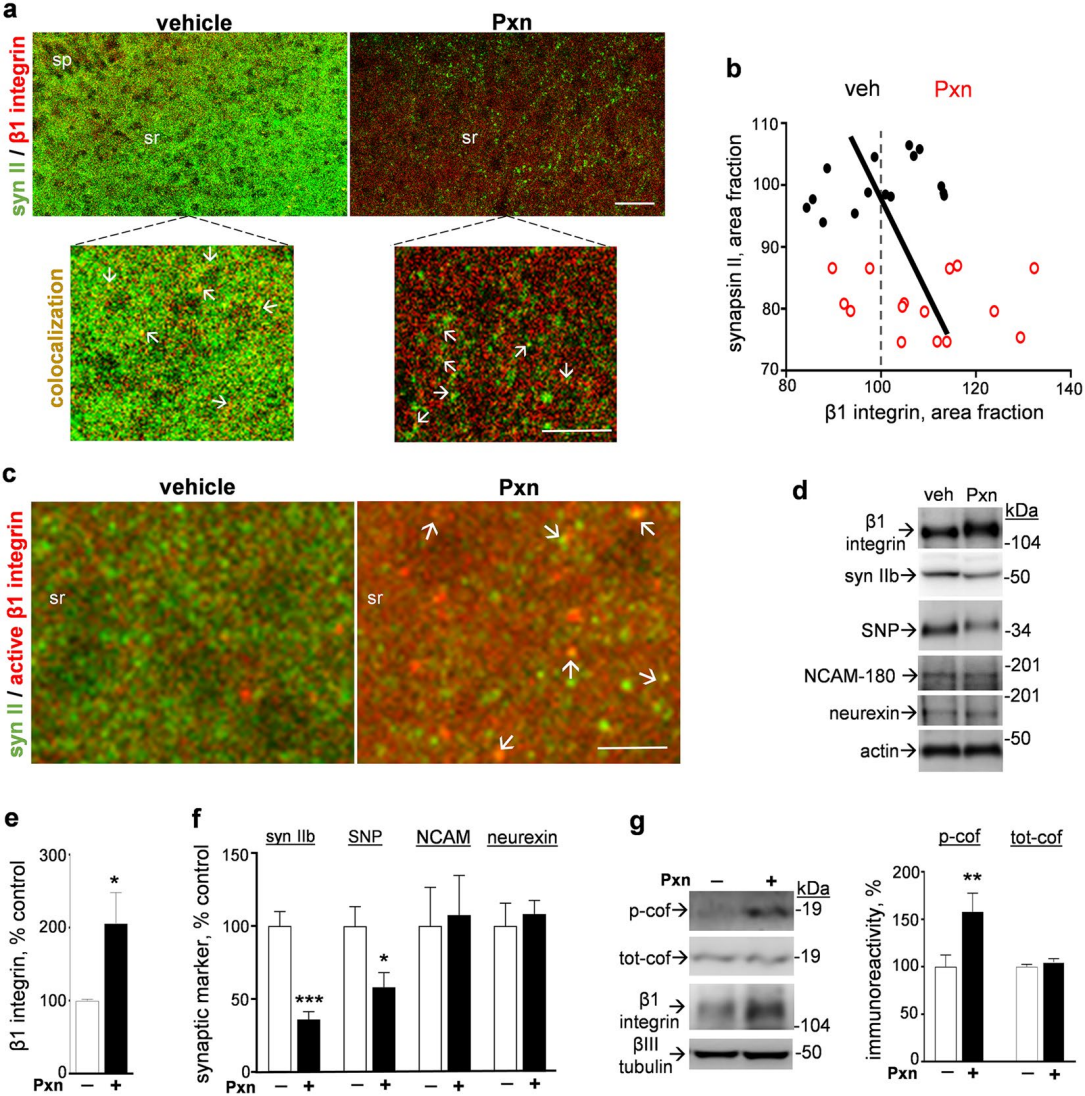
Selective β1 integrin response corresponds with synapsin II reduction in Pxn-treated slice cultures. Hippocampal slices were treated with vehicle or 200 µM Pxn for 24 h, assessed for synapsin II (syn II) and β1 integrin, and the merged images were processed for compressed maximal intensity projection (**a**, size bar: 50 µm). sp, stratum pyramidale; sr, stratum radiatum. The magnified views show punctate colocalization of synapsin II and β1 integrin staining in both control and Pxn slices (see arrows; size bar: 25 µm). Synapsin II and β1 integrin data sets were subjected to area fraction analyses, normalized to vehicle-treated slices, and plotted as a scattergram across dendritic region assessed (**b**). The relationship between the extent of synapsin II reduction and the extent of β1 integrin increase among vehicle control (veh, black dots) and Pxn-treated data (red circles) was assessed by linear regression (R = −0.822, p = 0.0410). Similar correspondence was found between the reduced synapsin II staining and increased punctate labeling for the active conformation of β1 integrin after Pxn treatment (**c**, size bar: 10 µm), with the two proteins often exhibiting colocalization (arrows). The slice groups were also analyzed by immunoblot to label β1 integrin, synapsin IIb, synaptophysin (SNP), NCAM-180, neurexin, and actin (**d**) (immunoblots are also presented in Supplementary Fig. S3). Positions of molecular weight standards are shown. Integrated optical density measures were determined and shown as mean percent of control ± SEM (**e**,**f**). Unpaired t-tests: *p < 0.05, ***p < 0.001. Immunoblot samples were also assessed for phosphorylated cofilin (p-cof), total cofilin (tot-cof), β1 integrin, and βIII tubulin (**g**) (see Supplementary Fig. S4). Integrated optical density measures were determined and shown as mean percent of control ± SEM (**g**). Unpaired t-tests: **p < 0.01.

The increased immunostaining of the β1 integrin subunit corresponded with reduced dendritic synapsin II labeling in Pxn-treated slices (Fig. 8a). In the high-magnification views shown in Fig. 8a, co-localization of β1 integrin and synapsin II was apparent in both control and Pxn-exposed tissue. Note that the extent of reduced synapsin II immunolabeling in defined dendritic areas significantly correlated with the extent of increased β1 integrin staining (Fig. 8b; R = −0.822, p < 0.05). Viewing the within-sample scattergram, only 35% of the β1 integrin measures from vehicle-treated samples plotted to the right of the established mean level (dashed line), whereas 73% of the measures from Pxn slices extended to levels greater than the control 100% β1 integrin mark. The negative relationship between β1 integrin and synapsin II levels is in contrast to the positive relationship between synapsin II and GluR1 staining in dendritic areas as mentioned above. The Pxn-mediated integrin response was confirmed using a monoclonal antibody that specifically recognizes the active conformation of β1 integrin (Fig. 8c). Arrows indicate the co-localization of active β1 integrin and synapsin II-positive puncta.

The integrity of central synapses is regulated by integrins and other adhesion molecules^53,54^. As shown in Fig. 8d (also see Supplementary Fig. S3), different classes of adhesion molecules were assessed using immunoblot samples and the Pxn-mediated increase in β1 integrin was selective as compared to two other synaptic adhesion components. The two-fold increase in β1 integrin immunoreactivity (Fig. 8e) was found in samples with significant declines in synaptic markers while also being strikingly divergent from the unaltered levels of the 162-kDa neurexin and the 180-kDa NCAM isoform (see Fig. 8d,f). As found with the comparative immunostaining in confocal images, the immunoblot measures of β1 integrin and the synapsin IIb isoform exhibited a strong correlation (R = −0.820, p = 0.002, n = 11).

The increase in β1 integrin was also associated with elevated levels of cofilin phosphorylated at serine 3 (Fig. 8g, n = 10–12 per condition; see Supplementary Fig. S4). Cofilin is an actin depolymerizing factor that is implicated, together with β1 integrin, in synaptic modulation and protection^45,55^. Total cofilin levels were unchanged as were βIII tubulin measures, whereas phosphorylated cofilin was significantly increased greater than 50% in the Pxn slices. This involvement of integrin–cofilin signaling links with the fact that β1 integrin co-localized with prominent staining of a synaptic marker whose overall immunolabeling is reduced in the Pxn-treated tissue (see Fig. 8c). Such results together suggest stochastic events of synaptic protection, perhaps encompassing a compensatory pathway that mitigates Pxn synaptotoxicity at some synapses.

**Figure 9:**
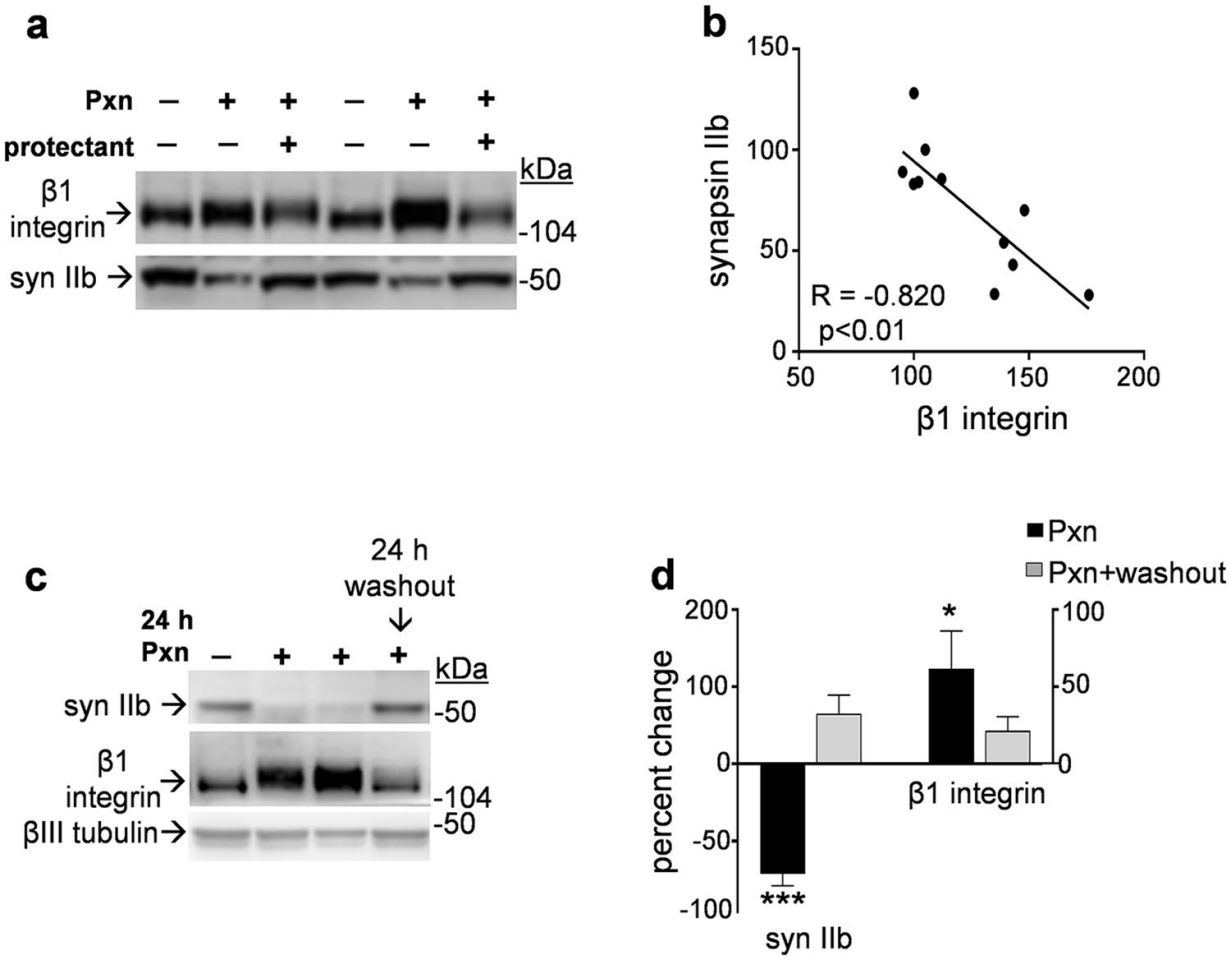
Attenuation of Pxn effects reveals a relationship between synaptotoxicity and the β1 integrin response. Slice cultures were pre-treated for 1 h with the protectant compound AM5206, and subsequently treated with Pxn and AM5206 for 24 h. The hippocampal slices were assessed for β1 integrin and synapsin IIb (syn IIb) by immunoblot and compared to control cultures (**a**). Levels of synapsin IIb were plotted against the within-sample measures of β1 integrin, with both antigens expressed as percent of control (**b**). Linear regression analysis was applied to the scatterplot (R = −0.820, p = 0.002). A subset of Pxn-treated slices exposed for 24 h were subjected to a washout procedure to remove the anticholinesterase, and the cultures were then incubated with fresh media for an additional 24 h. The harvested samples were assessed for synapsin IIb, β1 integrin, and βIII tubulin by immunoblot (**c**) (see Supplementary Figs S5 and S6). Immunoreactivities were normalized to their respective controls in order to plot percent change ± SEM (n = 12 Pxn samples, n = 5 Pxn + washout samples) for synapsin IIb and β1 integrin (**d**). Unpaired t-tests compared to respective controls: *p < 0.05, ***p < 0.001.

To test for a relationship between synaptotoxicity and the β1 integrin response, before toxin exposure slice cultures were pre-treated with a neuroprotectant^37^ whose effect greatly reduced the Pxn-mediated reduction in synapsin IIb (Fig. 9a and Supplementary Fig. S5). The diminished synaptotoxicity was associated with a reduced level of the Pxn-induced integrin response as previously found^37^, and evident across the immunoblot samples was a clear relationship between synapsin IIb and β1 integrin measures (Fig. 9b; p = 0.002). A similar correspondence was found between synaptic marker recovery and changes in β1 integrin when Pxn was washed out of the cultures and the tissue harvested 24 h later (Fig. 9c, see Supplementary Fig. S6). In the summarized results, the negative Pxn effect on synapsin IIb was eliminated and the β1 integrin measures returned to near control levels (Fig. 9d), further indicating that robust levels of synaptic decline are linked to the β1 integrin response.

## Discussion

This study took advantage of explant cultures prepared from the hippocampus, a brain region critical for learning and memory^26,33,49^, to examine the synapse-related changes after exposure to a deadly organophosphate. Pxn, a potent organophosphate pesticide, is approximately 70% as potent as the nerve agent sarin for the irreversible inhibition of acetylcholinesterase. Upon infusion into the hippocampal cultures, Pxn caused distinct reductions in synaptophysin, synapsin II, PSD-95, and GluR1, occurring in dendritic subfields well known for their involvement in communication networks for behavior and cognition. The synaptotoxic profiles were identified in the absence of any signs of neuronal degeneration. In addition, the study identified selective adhesion signaling in the exposed brain tissue, with the responses involving a synaptic adhesion molecule previously linked to the repair of neurons and to synapse maintenance in hippocampus^56–60^. This study highlights the distinctions and consequences of organophosphate toxicity in important brain circuitries.

The hippocampal slice cultures displayed remarkable alterations to pre- and postsynaptic markers after the Pxn exposure. The Pxn-induced synaptotoxicity was comprised of early and progressive declines in synaptophysin, synapsin IIb, and PSD-95, whereas a slower decay profile was exhibited by GluR1. This AMPA receptor subunit was unique in that the reduced staining for this marker was more apparent in the more distal dendrites stemming from pyramidal neurons, unlike the uniform weakening of synaptophysin-positive labeling of contacts surrounding pyramidal neurons and populating the dense dendritic fields. The Pxn effect on synaptic marker profiles was indeed found to be synapse-specific, noting no changes were found in the density or morphology of neurons, in the density or shape of DAPI-labeled nuclei, or in the density of dendritic labeling. In addition, NeuN, Nissl, and propidium iodide staining confirmed the absence of altered neuronal integrity in the stratum radiatum and molecular layer, the two zones that exhibit Pxn-mediated synaptotoxicity. Similar pathogenesis targeting synapses was previously described when hippocampal explants were exposed to the nerve agent soman^13^. Soman exposures resulted in markedly reduced synaptophysin labeling in the stratum radiatum, while the morphology and density of pyramidal neurons were unchanged. The current study showed that Pxn produces a significant reduction within the innervation pattern of synaptophysin staining, as well as correlative reductions in synapsin II and GluR1 labeling in both tissue and immunoblot samples.

It is noteworthy to point out the synaptic pathology produced by the widely used organophosphate insecticide chlorpyrifos. It caused subtle morphological changes to neuronal layers of the hippocampus *in vivo*, using a regimen that disrupted synapses and impaired cognition in the absence of systemic toxicity^61^. Prenatal exposure to such pesticides has been associated with significant reductions in childhood IQ^62–64^, thus Pxn, chlorpyrifos, and other organophosphate neurotoxicants need to be fully understood for their long-term effects on brain function. The cascade of events that influence synapses in Pxn-treated brain tissue leads to compromised levels of both pre- and postsynaptic components, and such compromise may underlie the brain anomalies in survivors of pesticide and nerve agent exposures.

The changes in synaptic components in Pxn-exposed brain slices may explain the neuronal dysfunction and memory impairment found in exposed animals^14,15^. Pxn distinctly changed the synaptic protein composition in the disparate hippocampal subfields. Such reductions in synaptic markers are signs of interference in synapse integrity and the precise molecular organization necessary for memory encoding. The synaptotoxicity found in dendritic zones may be linked to multiple alterations that disturb cellular homeostasis since, beyond its anticholinesterase action, Pxn also increases the release of glutamate in the hippocampus to cause enhanced glutamatergic activity and early expression of seizures^65,66^. Compared to another toxin that increases glutamatergic responses, synaptic markers were also found reduced when hippocampal slices were exposed to the excitotoxin kainic acid for 24 h^36^. However, in contrast to the Pxn results, the kainic acid-induced synaptic decline was associated with neuronal death, as also found for the toxic actions of trimethyltin^31^ and 3-nitropropionic acid^67^. Thus, the synapse-specific alterations mediated by Pxn appear to be an early and unique toxicological response in the brain.

Synaptic compromise is a precursor to many types of brain disorders that deteriorate cognitive ability. Pxn’s effect on synaptic proteins in the important hippocampal neuropil may reflect the pathogenic chain of events that leads to the weakening of synapses and, thus, disruption of cognition. Rats that survived Pxn treatment displayed increased anxiety and impaired recognition memory^21^. Also note that higher-order brain function was disrupted months after an acute exposure^19^. Interestingly, a correlation between CA1 hippocampal neuropathology and spatial memory impairment was found in rats treated with the organophosphate soman^68^. Thus, the types of symptoms, neurological problems, and behavioral changes in survivors of organophosphate exposure may stem from the early synaptotoxicity in the hippocampus and other cortical regions. Hippocampal neurons become vulnerable after experiencing brief or subtoxic insults^28,69,70^, likely explaining the increased risk to brain disorders in survivors of neurotoxin exposure. Such synaptic vulnerability was also found in hippocampal tissue treated with subtoxic levels of soman, after which the tissue exhibited enhanced synaptophysin loss in response to a brief, typically innocuous excitotoxin treatment^13^. Perhaps low-level organophosphate exposure, through pesticide applications or weaponized nerve agents, can enhance ones risk to excitotoxicity-related disorders including stroke and traumatic brain injury later in life. Note that the synaptic markers found reduced in Pxn-treated hippocampal slices were also found reduced by stroke-type excitotoxic insults^32,36^ and blast-induced neurotrauma^34^. Persistent synaptic compromise in hippocampal subfields likely plays a part in enhancing the risk for a subsequent disorder.

A number of studies provide evidence that organophosphate exposure can subsequently lead to neuropsychiatric conditions as well as an increased risk to develop Alzheimer’s disease, Parkinson’s disease, and other types of dementia^9,12,23^. The synapsin isoforms shown to be reduced in Pxn-treated hippocampal slices were also decreased by dementia-linked protein accumulation stress^39,71^. In addition, the major loss of PSD-95 in the Pxn-treated cultures warrants pointing out a report indicating that this synaptic marker is reduced in the hippocampus of subjects with amnestic mild cognitive impairment^72^, a condition thought to precede the development of dementia. These findings further support the idea that organophosphate exposure enhances one’s vulnerability to cognitive decline.

In contrast to the significant reductions exhibited by synaptic markers in Pxn-treated hippocampal slices, β1 integrin levels were found to be enhanced in dendritic fields and in the immunoblot samples of treated explants. Functional integrity of synapses is regulated by various adhesion molecules^53,54,73,74^ and the β1 integrin subunit stands out as being selectively upregulated by the Pxn exposure since two other adhesion molecules were unchanged. The Pxn-induced integrin response was particularly robust at punctate sites with strong, co-localized synaptic marker staining, indicative of protected synapses. In addition, since the substantial degree of Pxn-induced synaptotoxicity measured was not associated with the expected neurodegeneration, the β1 integrin involved may be part of a compensatory repair pathway as previously suggested^55,56,58,59^. The β1 integrin subunit is found in adult hippocampal neurons, is part of many integrin complexes in the brain, and the subunit and its integrin families have been localized to synaptic assemblies and sites of neuronal regeneration^46,47,75–78^. It is also noteworthy that integrin expression in neurons may be indirectly influenced by proliferative astrocytes. Previous studies found evidence that excitotoxic insults lead to enhanced astroglial expression of integrin-binding matrix proteins^79,80^. Thus, reactive astrocytes found after Pxn treatment may release extracellular matrix molecules that upregulate β1 integrin expression in neighboring neurons.

Integrin pathways are involved in the transduction of signals for actin reorganization events. The corresponding findings between induced β1 integrin responses and inactivation of the actin severing protein cofilin through phosphorylation suggest a pathway involved in stabilizing actin filaments and perhaps neuronal resiliency. In addition to β1 integrin–cofilin signaling found triggered by organophosphate exposure, previous reports indicate that integrin-driven actin polymerization is linked to the functional stabilization of hippocampal synapses^50^, while conditions that block cofilin phosphorylation and actin polymerization disrupt synaptic modulation^81^. Activated cofilin via reduced phosphorylation was implicated in synaptic decline produced by the Aβ42 peptide^55^, in contrast to reduced Aβ42 neurotoxicity found after the activation of β1 integrin^55,58^. Integrin signaling through focal adhesion kinase has also been linked to a neuroprotectant avenue^28,82^. Together, the different studies further suggest that integrin pathways are involved in neuronal responses to injury, perhaps slowing the synaptic marker declines to the gradual rates found in Pxn-exposed brain slices.

In summary, the altered synaptic markers due to Pxn exposure point to a type of toxicity that can have lasting effects, perhaps with few initial symptoms due to the absence of early neurodegeneration in the hippocampal explant model. Deterioration of synaptic integrity in a cognitive center like the hippocampus can be detrimental to many brain functions, as this cortical region subserves behavior, mood, and cognition. The extent of the identified synaptotoxicity may determine the level of consequences and vulnerabilities expressed later in exposure survivors. In addition to the established effects organophosphate exposure has on brain development and early cognitive ability, changes in maintenance factors for synaptic integrity can have lasting effects in the mature brain, slowly disrupting circuitries essential to higher order brain functions. Detecting early synaptic alterations may help determine treatments for vulnerable individuals who have come in contact with organophosphate-based insecticides or nerve agents before signs of behavioral and cognitive morbidities develop. Compensatory responses activated by organophosphate exposure may also play a role in determining the type and extent of neurological and behavioral alterations that occur in survivors. Better understanding of pathways that govern synaptic pathology will improve treatment strategies for organophosphate poisoning.

## Supplementary information

Supplementary figures
