# Supplementary material for: Early Synaptic Alterations and Selective Adhesion Signaling in Hippocampal Dendritic Zones Following Organophosphate Exposure
Source: Sci Rep. 2019 Apr 25;9:6532. doi: 10.1038/s41598-019-42934-z (PMC6484076; doi:10.1038/s41598-019-42934-z)

Supplementary information for:

Early Synaptic Alterations and Selective Adhesion Signaling in Hippocampal Dendritic Zones following Organophosphate Exposure

Karen L.G. Farizatto,[1] Michael F. Almeida,[1] Ronald T. Long,[1,2] and Ben A. Bahr[1-3]

[1]Biotechnology Research and Training Center, [2]Department of Biology, and [3]Department of Chemistry and Physics, University of North Carolina-Pembroke, Pembroke, North Carolina

Corresponding author:
B.A. Bahr, Biotech Center and Training Center,
1 University Drive, University of North Carolina – Pembroke, Pembroke NC 28372
Email: Bahr@uncp.edu

Farizatto et al., Supplementary Figure S1

Full-length blots from Figure 1g

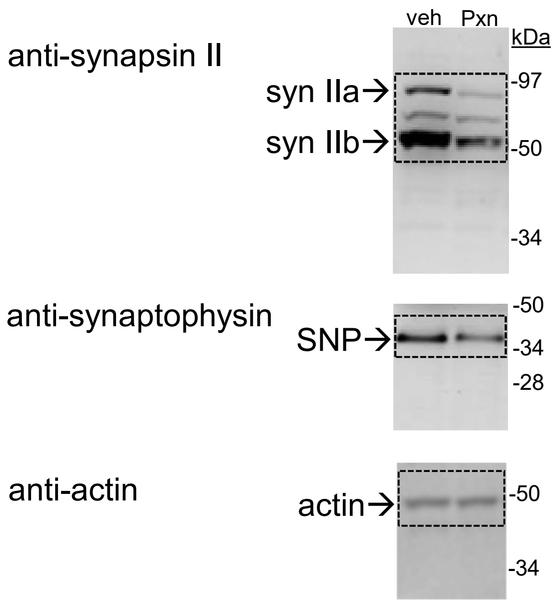

Farizatto et al., Supplementary Figure S2

Full-length blots from Figure 1h

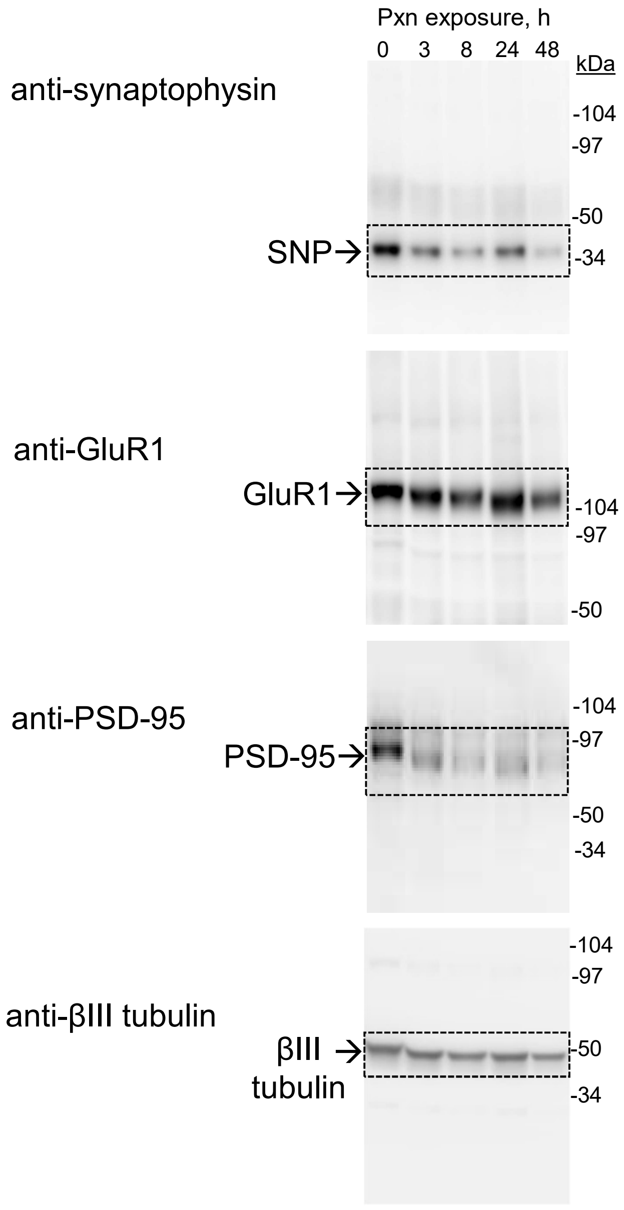

Farizatto et al., Supplementary Figure S3

Full-length blots from Figure 8d

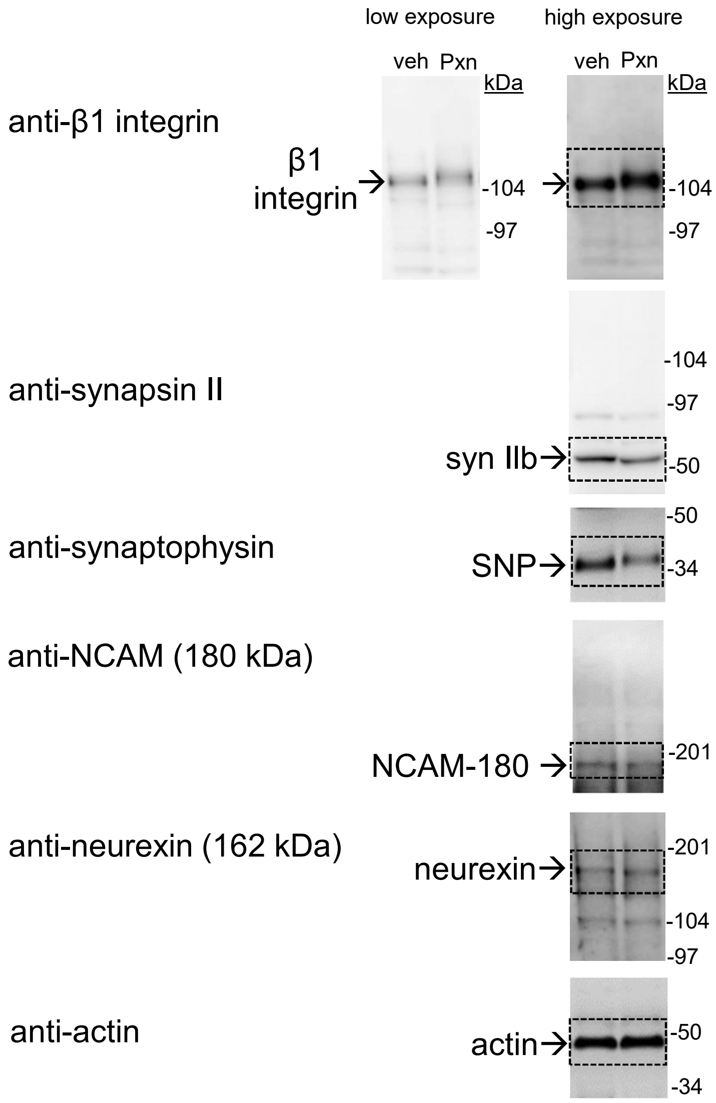

Farizatto et al., Supplementary Figure S4

Full-length blots from Figure 8g

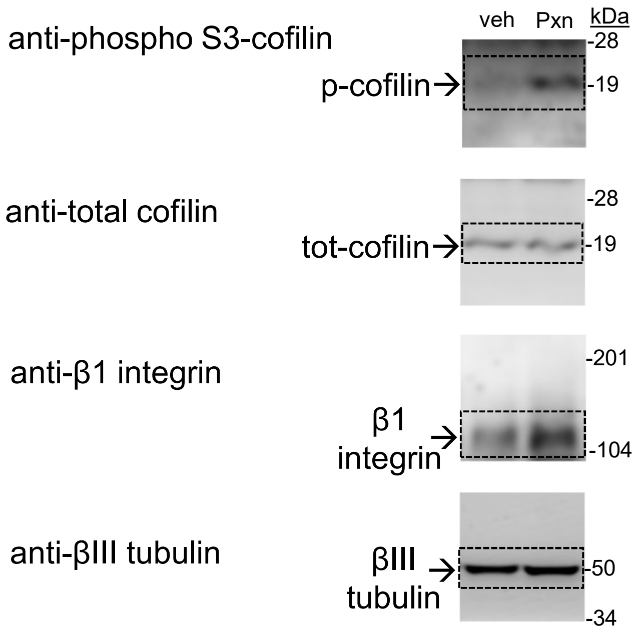

Farizatto et al., Supplementary Figure S5

Full-length blots from Figure 9a

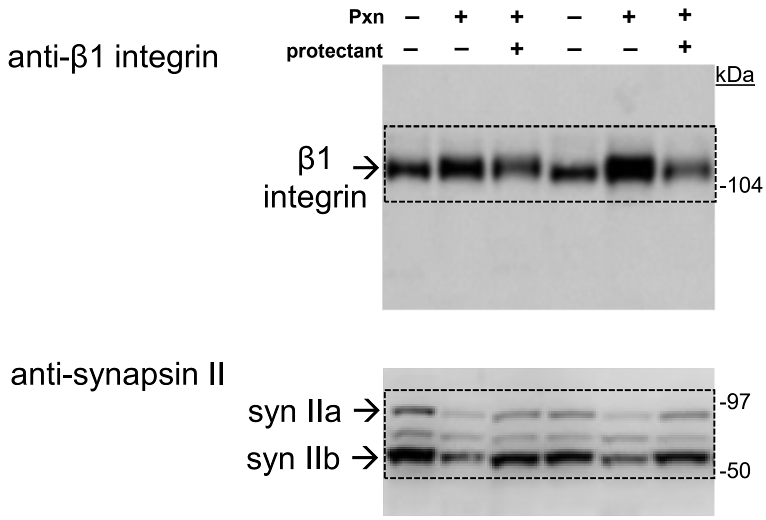

Farizatto et al., Supplementary Figure S6

Full-length blots from Figure 9c

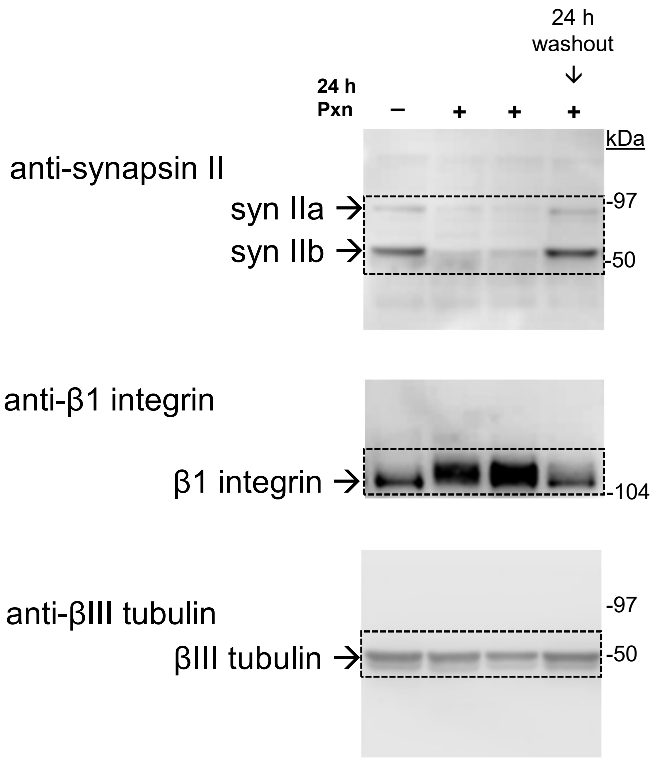

Supplement: Supplementary file 1 — Supplementary figures [file 41598_2019_42934_MOESM1_ESM.pdf]
